# Long-term metabolic and safety profiles of tenofovir alafenamide and tenofovir disoproxil fumarate in ART-naive people living with HIV: a multicenter retrospective study using a mixed-effects model

**DOI:** 10.1186/s40360-025-01003-0

**Published:** 2025-10-14

**Authors:** Oguz Karabay, Asli Vatan, Abdullah Ucar, Ilknur Yilmaz, Nurselin Can Balta, Arzu Kanturk, Ridvan Dumlu, Yasemin Cag, Hulya Caskurlu, Merve Tokgoz Şik, Zahide Asik Otman, Umay Balci, Seniha Senbayrak, Sila Akhan, Muge Toygar Deniz, Dilek Yekenkurul, Nevin Ince, Bekir Tunca, Tuba Damar Cakirca, Aliye Bastug, Unal Erkorkmaz, Ertugrul Guclu

**Affiliations:** 1 Department of Infectious Diseases and Clinical Microbiology, Faculty of Medicine, Sakarya University, Sakarya, Türkiye; 2 Medical Research and Development, ARGE. Technology Transfer Office of Sakarya University, Sakarya, Türkiye; 3 Department of Public Health, Faculty of Medicine, Sakarya University, Sakarya, Türkiye; 4 Department of Infectious Diseases and Clinical Microbiology, University of Health Sciences, Prof. Dr. Cemil Taşcıoğlu City Hospital, Istanbul, Türkiye; 5 Department of Infectious Diseases and Clinical Microbiology, Faculty of Medicine, Medipol University, Istanbul, Türkiye; 6 Department of Infectious Diseases and Clinical Microbiology, Faculty of Medicine, Istanbul Medeniyet University, Istanbul, Türkiye; 7 Department of Infectious Diseases and Clinical Microbiology, University of Health Sciences, Antalya Training and Research Hospital, Antalya, Türkiye; 8 Department of Infectious Diseases and Clinical Microbiology, Haydarpaşa Numune Training and Research Hospital, University of Health Sciences, Istanbul, Türkiye; 9 Department of Infectious Diseases and Clinical Microbiology, Faculty of Medicine, Kocaeli University, Kocaeli, Türkiye; 10 Department of Infectious Diseases and Clinical Microbiology, Faculty of Medicine, Düzce University, Düzce, Türkiye; 11 Department of Infectious Diseases and Clinical Microbiology, Şanlıurfa Training and Research Hospital, Şanlıurfa, Türkiye; 12 Department of Infectious Diseases and Clinical Microbiology, University of Health Science, Ankara Bilkent City Hospital, Ankara, Türkiye; 13 Department of Biostatistics, Faculty of Medicine, Sakarya University, Sakarya, Türkiye

**Keywords:** HIV, Tenofovir disoproxil fumarate, Tenofovir alafenamide, Metabolic effects, Lipid profile, Bone mineral density, Renal function, Mixed-effects model, Antiretroviral therapy

## Abstract

**Background:**

Tenofovir disoproxil fumarate (TDF) and tenofovir alafenamide (TAF) differ in their effects on renal, bone, and lipid parameters. However, long-term comparative data using robust statistical methods in real-world settings—especially among antiretroviral therapy (ART)-naive people living with HIV (PLWH)—are limited.

**Aims:**

This study compared the long-term metabolic and safety profiles of TDF versus TAF in ART-naive PLWH, using mixed-effects modeling to inform clinical decisions.

**Methods:**

A multicenter retrospective cohort study was conducted across 10 HIV centers in Türkiye from 2018 to 2022. The study included 540 ART-naive PLWH aged ≥ 18 years, treated with TDF- or TAF-based regimens for ≥ 12 months, with ≥ 24 months follow-up. Primary endpoints were changes in renal function (eGFR), bone mineral density (T-scores), and lipid profiles over 48 months. Mixed-effects regression models adjusted for baseline demographics, comorbidities, and concomitant medications.

**Results:**

Both TAF (*n* = 197) and TDF (*n* = 343) showed similar virological suppression and CD4 + recovery, with no significant differences in HIV RNA (*p* > 0.24) or CD4 + counts (*p* > 0.54). At 24 months, TAF led to greater increases in total cholesterol, non-HDL cholesterol, LDL cholesterol, and triglycerides (*p* ≤ 0.039), though differences were not significant at 48 months. Phosphorus levels were similar (*p* > 0.2), but eGFR declined more with TAF at 48 months (*p* < 0.001). Overall changes in bone mineral density were comparable (*p* > 0.18). GGT increases at 24 months were greater with TAF in both sexes; the clinical significance is uncertain and warrants monitoring.

**Conclusion:**

Over 48 months, TAF and TDF achieved equivalent virological and immunological responses. In this real-world retrospective cohort, TAF was linked to short-term lipid increases and a greater eGFR decline, while bone density changes were similar overall. Given baseline imbalances, unmeasured confounding (including concomitant medications and regimen components), and multiple testing, these findings should be interpreted cautiously and considered hypothesis-generating. Antiretroviral choice should be individualized.

**Supplementary Information:**

The online version contains supplementary material available at 10.1186/s40360-025-01003-0.

## Introduction

Despite advances in antiretroviral therapy (ART), people living with HIV (PLWH) still face long-term metabolic and safety challenges from their treatments, contributing to increased morbidity and mortality [1]. Among nucleoside reverse transcriptase inhibitors, tenofovir disoproxil fumarate (TDF) and tenofovir alafenamide (TAF) are widely used as part of first-line ART combinations [2]. While both agents are effective in suppressing viral replication, their safety profiles differ substantially. TDF has been associated with bone mineral density loss and impaired renal function, leading to increased risks of osteoporosis and renal complications. In contrast, TAF is considered safer for bone and kidney health but has been linked to unfavorable changes in lipid profiles, potentially increasing cardiovascular morbidity and mortality [3].

Although randomized controlled trials and meta-analyses have demonstrated comparable virological efficacy for TDF and TAF, real-world data on their long-term metabolic and safety outcomes—including their impact on morbidity and mortality—especially in ART-naive populations, remain limited [4]. Moreover, most existing studies do not adequately account for inter-individual variability or the complex, longitudinal nature of metabolic changes observed in clinical practice [5, 6].

Personalized treatment approaches that consider genetic, biological, and environmental factors are essential to optimize outcomes and minimize adverse effects in PLWH. However, studies utilizing advanced statistical methods such as mixed-effects models to address these complexities are scarce [7].

To address this gap, our multicenter retrospective study uses real-world data and mixed-effects modeling to comprehensively compare the long-term metabolic and safety profiles of TDF and TAF in ART-naive PLWH. Our findings aim to inform personalized treatment strategies and optimize long-term care for this population.

## Methods

### Study population and design

This multicenter retrospective cohort study was conducted across 10 HIV treatment centers in Türkiye between January 2018-December 2022. A total of 540 patients receiving integrase inhibitor-based TDF regimens (TDF/FTC + DTG, TDF/FTC/c/EVG, TDF/FTC + RAL) or TAF regimens (TAF/FTC/BIC, TAF/FTC/c/EVG) were included from 10 centers in Türkiye. The medical records of the patients were reviewed retrospectively. A total of 540 patients receiving integrase inhibitor-based TDF regimens or TAF regimens were included from these participating centers. This study included patients from 10 specialized HIV treatment centers across Türkiye: Sakarya University Faculty of Medicine, Prof. Dr. Cemil Taşcıoğlu City Hospital (Istanbul), Medipol University (Istanbul), Istanbul Medeniyet University, Antalya Training and Research Hospital, Haydarpaşa Numune Training and Research Hospital (Istanbul), Kocaeli University, Düzce University, Şanlıurfa Training and Research Hospital, and Ankara Bilkent City Hospital. Several investigators had affiliations with multiple institutions and contributed patients from 10 centers within their networks, ensuring comprehensive geographic representation across Türkiye.

### Sample size considerations

Given the retrospective design of this study, a formal a priori sample size calculation was not performed. Instead, all eligible patients meeting the inclusion criteria from the participating centers were included between January 2018 and December 2022. A post hoc power analysis was conducted for non-significant findings to assess the adequacy of statistical power. Post-hoc power analysis with f² = 0.02 (small effect size), α = 0.05, and *n* = 540 yielded 84.36% power for detecting differences between treatment groups in the primary mixed-effects model. While this suggests adequate power for small effects, the post-hoc nature of this calculation limits its interpretive value for study planning. Furthermore, the large sample size (*n* = 540) and extended follow-up period (up to 48 months) allowed for robust mixed-effects modeling to detect meaningful longitudinal differences between the TDF and TAF groups. This pragmatic design ensured comprehensive inclusion of real-world data while maintaining sufficient statistical rigor.

### Patient selection process

All patients meeting the predefined eligibility criteria were systematically identified through electronic medical records review at each participating center. The selection process ensured comprehensive inclusion of eligible patients while maintaining strict adherence to inclusion and exclusion criteria to minimize selection bias.

### Inclusion criteria

Participants were required to meet all predefined eligibility criteria for study inclusion. All participants must be aged 18 years or older at treatment initiation and have confirmed HIV-1 infection documented by both ELISA and Western Blot tests. Patients must be antiretroviral therapy (ART)-naïve at baseline with no prior exposure to any antiretroviral medications. Eligible participants were required to be receiving integrase inhibitor-based regimens containing either TDF-based combinations (TDF/FTC + DTG, TDF/FTC/c/EVG, or TDF/FTC + RAL) or TAF-based combinations (TAF/FTC/BIC or TAF/FTC/c/EVG). Patients must have completed a minimum of 12 months of continuous treatment with their assigned regimen and have a minimum of 24 months of documented clinical follow-up from treatment initiation. Additional requirements included available baseline measurements and at least two follow-up assessments for primary endpoints, treatment at one of the 10 participating HIV treatment centers in Türkiye between January 2018 and December 2022. Data completeness for key laboratory and clinical parameters was essential for inclusion in the final analysis.

### Exclusion criteria

Patients will be excluded if they have been followed up for less than 24 months from treatment initiation, are under 18 years of age, or have any prior exposure to antiretroviral therapy.

### Data collection

Data for the study were obtained from outpatient follow-up forms and hospital databases used for HIV/AIDS patients. Demographic, laboratory, and clinical information of the patients was collected retrospectively. The recorded data encompassed demographic information including age, sex, weight, height, and body mass index (BMI). Comorbidity data captured the presence of diabetes mellitus, hypertension, chronic kidney disease, coronary artery disease, and hyperlipidemia. Treatment information documented the initiated ART regimen and other medications used throughout the study period.

Laboratory and clinical parameters included HIV RNA levels, CD4 + T lymphocyte count, creatinine, estimated glomerular filtration rate (eGFR), total cholesterol, low-density lipoprotein (LDL), high-density lipoprotein (HDL), and triglyceride levels. Bone mineral density (BMD) measurements of the lumbar spine and femoral neck were obtained using dual-energy X-ray absorptiometry (DEXA). These parameters were systematically recorded at baseline (month 0) and at months 12, 24, 36, and 48 of follow-up. For patients who underwent ART regimen changes during treatment, the specific reasons for these modifications were documented and obtained from follow-up forms to ensure comprehensive tracking of treatment patterns [8].

Osteoporosis diagnosis was established according to the European AIDS Clinical Society guidelines. For postmenopausal women and men aged 50 years or older, osteoporosis was defined as a bone mineral density (BMD) T-score of -2.5 or lower at either the femoral neck or lumbar spine. In premenopausal women and men under 50 years of age, osteoporosis was defined as a BMD Z-score of -2 or lower at either the femoral neck or lumbar spine [9].

### Concomitant medication use

A key limitation of this retrospective study is the lack of systematic documentation on concurrent medications known to significantly impact metabolic parameters. These include lipid-lowering agents (such as statins, fibrates, and ezetimibe), antidiabetic drugs (including metformin, insulin, and SGLT2 inhibitors), antihypertensive therapies (ACE inhibitors, ARBs, diuretics), as well as weight management medications and supplements influencing lipid metabolism. This omission represents a critical methodological weakness that substantially undermines the interpretability of our metabolic results, given that these medications can induce changes in lipid profiles comparable to or greater than the differences observed between treatment groups.

### Endpoints

#### Primary endpoint

Comparison of the effects of integrase inhibitor-based TDF and TAF regimens on bone metabolism lipid profile renal function

#### Secondary endpoint

Comparison of the frequency of adverse effects associated with treatment combinations between the groups.

### Statistical analysis

For preliminary statistical analyses, the independent samples t-test was used for parametric variables, the Mann–Whitney U test was used for non-parametric variables, and the chi-square test was applied for comparisons of categorical variables. The results were presented as frequencies, means, and percentages. The p-value threshold for statistical significance was set at *p* < 0.05.

The FDA recommends Mixed-Effect Regression (MER) analysis in comparative drug studies, especially in data sets based on longitudinal measurements [10]. In MER analysis, comparisons between different groups are made by comparing the trends of measurements in different groups over time, not by comparing within-group or between-group measurement averages.

MER analysis effectively handles missing data points within the dataset by utilizing all available measurements from each participant, even when some time points are missing. This approach maximizes the use of available data and ensures efficient incorporation of the entire dataset into the analysis. Additionally, MER analysis compares regression slopes between groups over time rather than simple averages at specific measurement points, which automatically controls for baseline differences between groups and individuals. Among all variables analyzed, HIV RNA measurements required logarithmic transformation due to their high numerical values, and were therefore included in the analysis as natural logarithm values [11].

In this study, Linear Mixed-Effects Regression analysis was performed using SPSS v24. Patient IDs were specified as random effects to account for repeated measurements, while group assignment (TAF vs. TDF) and measurement time were included as fixed effects.

Prior to model fitting, preliminary analyses were conducted to evaluate potential baseline imbalances between groups. Age, gender, and BMI were compared using the chi-square test and Mann–Whitney U test, as appropriate. No significant difference was found for BMI (*p* = 0.327); however, both age (*p* = 0.025) and sex (*p* = 0.005) differed significantly between the TAF and TDF groups. Therefore, age and gender were included in the mixed-effects model as covariates to control for potential confounding.

In the model specification, group and sex were included as categorical fixed factors, while measurement time and age were included as continuous covariates. The variance components structure was selected as the covariance type, and the Restricted Maximum Likelihood method was used for parameter estimation.

Laboratory values (HIV_RNA_Log, CD4, Creatinine, GFR, Phosphor, Total Cholesterol, HDL, Non-HDL Cholesterol, LDL, Triglyceride, Spine BMD, Femur BMD) were determined as dependent variables of the study. Treatment group (TAF, TDF) and measurement time (0-12-24-36-48th months) were used as independent variables.

Two models were constructed in the study. The first model includes 5-time measurements from baseline to the 48th month, i.e., the whole data set. However, it was considered that different confounders might weaken the model as the measurement period was prolonged, so a 3-time model (Model 2: baseline to 24th month) was applied too. The differences between the TAF & TDF groups in both models are presented in Table 2.

## Results

A total of 540 naive individuals were included in the study, with 343 participants in the TDF group and 197 in the TAF group at baseline. The mean age of participants was 42.2 years in the TDF group and 40.1 years in the TAF group, with an overall mean age of 41.4 years. The proportion of female participants was 16.6% (*n* = 57) in the TDF group and 8.1% (*n* = 16) in the TAF group. The mean duration since HIV diagnosis was 4.9 years in the TDF group and 4.2 years in the TAF group. Similarly, the mean body mass index (BMI) was slightly higher in the TDF group (24.28 kg/m²) compared to the TAF group (23.90 kg/m²). The baseline characteristics of the participants are summarized in Table 1.

**Fig. 1:**
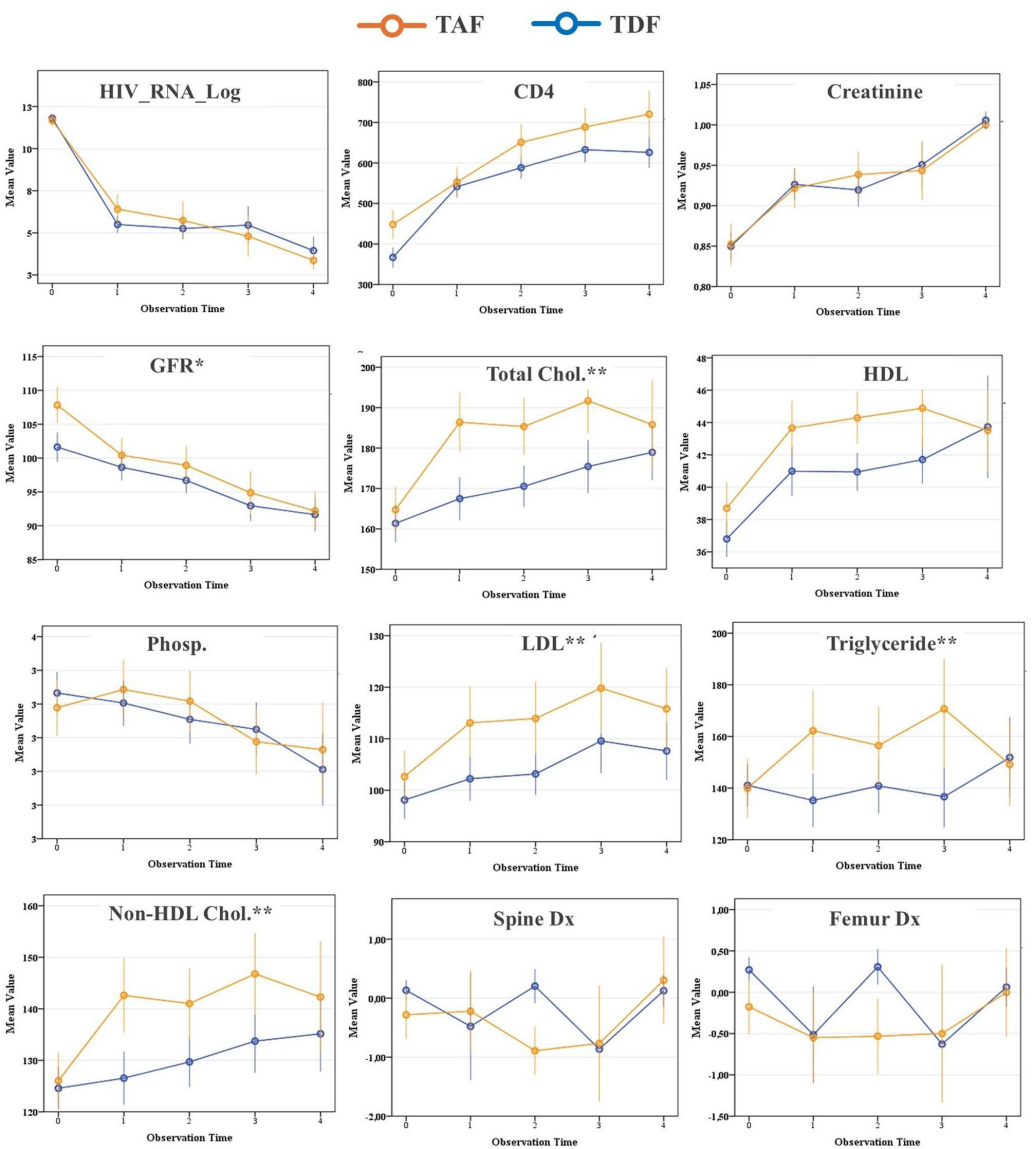
Longitudinal changes in metabolic, renal, and bone parameters among ART-Naive PLWH treated with Tenofovir Alafenamide (TAF) or Tenofovir Disoproxil Fumarate (TDF). The term “observation time” refers to the measurement time. *There is a significant difference between the two groups over the 4-year follow-up. **There is a significant difference between the two groups over the 2-year follow-up

Figure 1 shows the distribution of mean measurement values for TAF and TDF groups over time. Compared at baseline, the difference in HIV_RNA, Creatinine, Phosphorus, Total Cholesterol, HDL, Non_HDL_Cholesterol, LDL, and Triglyceride were not statistically significant (*p* > 0.05). In contrast, the difference in other variables (CD4, GFR, Spine BMD, Femur BMD) was significant.

Table 2 presents the linear MER analysis results comparing the effects of TDF and TAF regimens on clinical and biochemical parameters. The analysis covers two time intervals: Model 1 (baseline to 48 months) and Model 2 (baseline to 24 months). The table provides the slopes for each variable in both groups, the differences in slopes between the groups, and the statistical significance of these differences.

A significant increase in CD4 + T cell counts was observed over time within both the TDF and TAF groups; however, when comparing the two groups directly, no significant difference in the magnitude of CD4 + T cell increase was found in either Model 1 or Model 2 (difference in Model 1: 0.8, *p* > 0.05; difference in Model 2: 7.1, *p* > 0.05), indicating similar immunological recovery between the groups.

While there was no significant difference between the groups for non-HDL cholesterol in Model 1, in Model 2, the slope changed by 7.583 in the TAF group and 2.377 in the TDF group, with a statistically significant between-group difference (-5.206, *p* = 0.001). Similarly, there was no significant difference between the groups for total cholesterol in Model 1. The slope changed by 10.287 in the TAF group and 4.213 in the TDF group, with a statistically significant between-group difference (-6.074, *p* = 0.001). In model 2, the LDL slope changed by 5.691 in the TAF group and 2.138 in the TDF group, with a statistically significant between-group difference (-3.552, *p* = 0.039). Similarly, the triglyceride slope changed by 10.042 in the TAF group and − 0.991 in the TDF group, with a statistically significant between-group difference (-11.033, *p* = 0.01).

When subgroup analyses were performed according to sex for the variables for which no difference was found between the groups in the analyses, for example, the Spine BMD slope for female patients in Model 1 was 0.602 in the TAF group. It was − 0.039 in the TDF group (Table 4). There was a statistically significant between-group difference (-0.640, *p* = 0.001). HDL slope for female patients in Model 2 was 5.921 in the TAF group and 1.86 in the TDF group, with a statistically significant between-group difference (-4.059, *p* = 0.024).

**Table 1:** Summary of baseline characteristics of Participants*

Characteristic*	TDF (*N* = 343)	TAF (*N* = 197)	Total
Female	57 (16.6)	16 (8.1)	73
Male	286 (83.4)	181 (91.9)	467
Age, years – mean (SD)	42.2 (11.7)	40.1 (12.0)	41.4 (11.9)
Diagnosis Time, years - mean (SD)	4.9 (2.0)	4.2 (1.8)	4.7 (2)
BMI kg/m^2^ - mean (SD)	24.3 (4.36)	23.9 (3.78)	24.2 (4.17)

*Baseline means the observation time = t0

N: count value

SD: Standard Deviation

TAF: tenofovir alafenamide

TDF: tenofovir disoproxil fumarate,

Sex values are presented as n (%)

**Table 2:** Comparison of TAF and TDF groups using linear mixed effects model analysis

Variable	Model 1: Baseline to 48th Month*				Model 2: Baseline to 24th Month*			
	TAF Slope**	TDF Slope**	Between-group difference in slope	*p* value	TAF Slope**	TDF Slope**	Between-group difference in slope	*p* value
HIV RNA Logarithmic	-2,517	-2,717	-0,200	0,357	-3,590	-3,986	-0,396	0,242
CD4+	82,324	81,553	-0,771	0,902	115,717	122,704	6,988	0,543
Creatinine	0,034	0,034	0,000	0,992	0,043	0,037	-0,006	0,401
**Glomerular Filtration Rate**	**-3**,**566**	**-2**,**169**	**1**,**397**	**0**,**000**	-4,360	-2,949	1,411	0,063
Phosphorus	-0,028	-0,049	-0,022	0,214	0,007	-0,033	-0,040	0,219
Total Cholesterol	4,497	3,033	-1,464	0,139	**10**,**262**	**4**,**189**	**-6**,**073**	**0**,**001**
High-Density Lipoprotein Cholesterol	1,542	1,435	-0,106	0,758	2,740	1,910	-0,830	0,112
Non-HDL Cholesterol	2,960	1,628	-1,332	0,169	**7**,**556**	**2**,**341**	**-5**,**215**	**0**,**001**
Low-Density Lipoprotein Cholesterol	2,822	2,076	-0,747	0,441	**5**,**716**	**2**,**120**	**-3**,**596**	**0**,**039**
Triglyceride	3,586	0,014	-3,572	0,121	**9**,**974**	**-1**,**026**	**-11**,**000**	**0**,**010**
Spine Bone Mineral Density	-0,047	-0,048	-0,001	0,983	-0,225	-0,098	0,128	0,225
Femur Bone Mineral Density	-0,004	-0,071	-0,068	0,181	-0,110	-0,092	0,017	0,811

Fixed Effects: TAF & TDF Groups, Measurement Times, Age, Gender. Random Effects: Patient IDs (intercepts). TAF: tenofovir alafenamide, TDF: tenofovir disoproxil fumarate

*Baseline: measurement at month 0 (t0). Measurement times: 0-12-24-36-48th months. Model 1 includes 0-12-24-36-48th measurements and Model 2 includes 0-12-24th measurements. ** A positive value indicates increasing slope, and negative slope indicates decreasing slope

**Table 3:** Comparative analysis of Tenofovir Alafenamide (TAF) and Tenofovir disoproxil fumarate (TDF): changes in laboratory parameters over 24 and 48 months by age group

Variable	Model 1: Baseline to 48th Month*						Model 2: Baseline to 24th Month*			
	Age	TAF Slope**	TDF Slope**	The between-group difference in slope	p value		TAF Slope**	TDF Slope**	The between-group difference in slope	p value
Alanine Aminotransferase	20–49	-0,335	-1,542	-1,207	0,07		-1,346	-3,121	-1,776	0,154
	50+	0,117	-3,158	-3,274	0,096		0,859	-5,269	-6,128	0,182
CD4	20–49	82,362	82,669	0,307	0,966		119,572	122,515	2,943	0,821
	50+	82,988	77,276	-5,712	0,642		101,716	123,047	21,33	0,38
Creatinine	20–49	0,034	0,034	0	0,991		0,043	0,034	-0,009	0,241
	50+	0,035	0,034	-0,001	0,947		0,046	0,051	0,004	0,791
Femur Bone Mineral Density	20–49	-0,049	-0,073	-0,024	0,654		-0,075	-0,088	-0,013	0,846
	50+	0,071	-0,06	-0,131	0,301		-0,23	-0,105	0,125	0,603
Glomerular Filtration Rate	**20–49**	**-3**,**83**	**-2**,**148**	**1**,**682**	**0**		**-4**,**483**	**-2**,**77**	**1**,**713**	**0**,**047**
	50+	-2,895	-2,438	0,457	0,575		-4,001	-3,621	0,38	0,815
Gamma-Glutamyl Transferase	20–49	-0,444	-1,356	-0,912	0,329		**1**,**041**	**-3**,**336**	**-4**,**377**	**0**,**018**
	50+	1,289	-2,961	-4,251	0,097		4,366	-3,66	-8,026	0,138
High-Density Lipoprotein Cholesterol	20–49	1,48	1,383	-0,097	0,815		2,378	1,715	-0,662	0,242
	50+	1,757	1,612	-0,145	0,808		3,981	2,542	-1,439	0,246
HIV_RNA	20–49	-366.037,89	-296.602,58	69.435,31	0,689		-819.905,58	-579.400,65	240.504,94	0,457
	50+	-276.717,88	-1.121.298,56	-844.580,68	0,235		-657.297,82	-1.240.364,92	-583.067,10	0,454
Low-Density Lipoprotein Cholesterol	20–49	2,666	1,571	-1,095	0,219		4,028	1,413	-2,615	0,089
	50+	2,992	3,6	0,608	0,83		11,171	3,511	-7,66	0,165
Non-HDL Cholesterol	**20–49**	**3**,**859**	**1**,**176**	**-2**,**683**	**0**,**013**		**7**,**243**	**1**,**693**	**-5**,**55**	**0**,**001**
	50+	0,459	3,299	2,84	0,179		8,348	4,233	-4,115	0,325
Phosphorus	20–49	-0,037	-0,05	-0,014	0,507		-0,02	-0,037	-0,016	0,664
	50+	0,001	-0,05	-0,051	0,137		**0**,**103**	**-0**,**023**	**-0**,**126**	**0**,**045**
Spine Bone Mineral Density	20–49	-0,021	-0,061	-0,04	0,545		-0,108	-0,106	0,002	0,982
	50+	-0,201	0,009	0,21	0,139		**-1**,**494**	**-0**,**066**	**1**,**429**	**0**,**0001**
Total Cholesterol	**20–49**	**5**,**309**	**2**,**492**	**-2**,**817**	**0**,**009**		**9**,**573**	**3**,**332**	**-6**,**24**	**0**,**001**
	50+	2,224	4,919	2,695	0,239		12,302	6,722	-5,58	0,227
Triglycerides	20–49	4,306	0,274	-4,032	0,114		**11**,**574**	**0**,**392**	**-11**,**182**	**0**,**015**
	50+	1,606	0,025	-1,581	0,76		4,684	-5,414	-10,099	0,334
Weight	20–49	0,895	0,774	-0,121	0,485		0,994	0,809	-0,185	0,514
	50+	0,917	0,913	-0,004	0,988		1,117	0,931	-0,186	0,72

Slope values: Rate of change over time

Negative difference: Favors TAF

Positive difference: Favors TDF

Bold p-values: Statistically significant (*p* < 0.05)

**Table 4:** Sex-Stratified comparison of Tenofovir Alafenamide (TAF) and Tenofovir disoproxil fumarate (TDF): changes in laboratory parameters over 24 and 48 months

Parameter	Model 1: Baseline to 48th Month*						Model 2: Baseline to 24th Month*			
	Sex	TAF Slope	TDF Slope	Difference	p-value		TAF Slope	TDF Slope	Difference	p-value
Alanine Aminotransferase	F	1.129	-2.146	-3.276	0,136		1.700	-4.679	-6.379	0,165
	**M**	**-333**	**-1.869**	**-1.536**	**0**,**037**		-1.139	-3.467	-2.328	0,120
CD4+	F	94.301	89.906	-4.395	0,813		136.551	138.349	1.798	0,958
	M	81.023	79.720	-1.303	0,845		113.617	119.392	5.775	0,639
Creatinine	F	52	56	4	0,793		31	35	4	0,839
	M	33	29	-3	0,456		45	38	-7	0,369
Femur Bone Mineral Density	F	164	-61	-225	0,366		-940	-123	817	0,092
	M	-21	-72	-51	0,303		-98	-82	16	0,836
Glomerular Filtration Rate	F	-3.257	-2.760	497	0,707		-1.866	-2.958	-1.092	0,640
	**M**	**-3.599**	**-2.038**	**1.561**	**0**,**000**		-4.609	-2.959	1.651	0,042
Gamma-Glutamyl Transferase	F	3.765	-3.323	-7.087	0,113		**12.695**	**-6.719**	**-19.414**	**0**,**033**
	M	-249	-1.508	-1.259	0,164		**1.061**	**-2.947**	**-4.007**	**0**,**027**
High-Density Lipoprotein Cholesterol	F	2.722	1.804	-917	0,379		**5.951**	**1.850**	**-4.101**	**0**,**024**
	M	1.440	1.354	-86	0,817		2.484	1.922	-562	0,302
HIV RNA Logarithmic	F	-2.707	-2.476	232	0,787		-2.904	-2.536	368	0,755
	M	-2.505	-2.746	-241	0,286		-3.644	-4.255	-611	0,082
HIV RNA Viral Load	F	-292,336.435	-398,945.898	-106,609.463	0,853		-374,440.621	-473,188.104	-98,747.484	0,901
	M	-338,061.891	-451,055.537	-112,993.646	0,552		-742,842.442	-802,785.126	-59,942.684	0,860
Low-Density Lipoprotein Cholesterol	F	-2.200	509	2.710	0,361		6.258	-418	-6.676	0,201
	M	3.228	2.416	-811	0,434		5.669	2.592	-3.077	0,099
Non-HDL Cholesterol	F	-3.073	-722	2.351	0,491		10.669	-733	-11.402	0,077
	M	3.433	2.153	-1.280	0,205		**7.219**	**2.982**	**-4.237**	**0**,**011**
Phosphorus	F	31	-10	-40	0,468		112	-18	-129	0,179
	M	-33	-58	-26	0,172		-4	-36	-33	0,348
Spine Bone Mineral Density	**F**	**578**	**-39**	**-617**	**0**,**042**		91	-116	-208	0,748
	M	-80	-54	27	0,680		-241	-80	161	0,160
Total Cholesterol	F	-324	1.150	1.473	0,700		**16.370**	**1.195**	**-15.175**	**0**,**032**
	M	4.865	3.466	-1.399	0,168		**9.648**	**4.800**	**-4.848**	**0**,**007**
Triglycerides	F	-1.527	-7.080	-5.554	0,381		10.356	-12.888	-23.245	0,068
	M	3.954	1.519	-2.435	0,329		9.837	1.474	-8.363	0,067
Weight	F	401	1.233	832	0,127		763	1.434	671	0,492
	M	956	712	-243	0,112		1.052	706	-346	0,160

Slope values: Rate of change over time

Negative difference: Favors TAF

Positive difference: Favors TDF

Bold p-values: Statistically significant (*p* < 0.05)

F = female, M = male

## Discussion

Our findings reveal that while TDF and TAF-based regimens offer comparable virological efficacy in the management of HIV infection, they differ significantly in terms of metabolic, biochemical, and bone health effects. In this study, the long-term metabolic effects of TDF and TAF-based regimens in PLWH who were followed for 48 months across 10 centers in Türkiye were compared using MER analysis. Given the longitudinal nature of the metabolic data, the FDA-recommended MER analysis was employed. This method allows for evaluating trends over time rather than simple comparisons of means at specific time points. Additionally, it accounts for baseline inter-individual variability, enabling a more precise and reliable data analysis. MER analysis is also robust in handling missing data, making it a powerful statistical ability for evaluating longitudinal datasets.

The findings demonstrated significant improvements in both treatment regimens’ viral suppression and CD4 + T cell counts. Both TDF and TAF-based regimens achieved equivalent virological suppression and CD4 + T cell recovery, with no significant between-group differences, consistent with established literature. Treatment selection should therefore focus on safety and tolerability profiles [12, 13].

Regarding renal function, our study revealed complex and somewhat unexpected findings that warrant careful interpretation. While no significant differences were observed in creatinine or phosphorus levels between the TAF and TDF groups, the estimated glomerular filtration rate (eGFR) declined significantly more in the TAF group. This finding contradicts established literature, which generally supports TAF’s superior renal safety profile [14]. This apparent paradox is likely attributable to channeling bias inherent in our retrospective study design. In clinical practice, TAF-containing regimens are preferentially prescribed to patients with baseline renal impairment or higher renal risk due to their improved safety profile. Consequently, the TAF group may represent a population with worse baseline renal function and greater susceptibility to eGFR decline. This channeling bias introduces heterogeneity between treatment groups in terms of baseline renal function and comorbidities, suggesting that the observed eGFR differences may reflect patient selection rather than direct drug effects. Although our mixed-effects models adjusted for baseline renal function and comorbidities, the retrospective nature of the data and potential unmeasured confounders limit the ability to fully eliminate this bias. Therefore, the eGFR decline in the TAF group should be interpreted cautiously as a likely consequence of higher-risk patients being channeled into this treatment arm rather than evidence of TAF-induced nephrotoxicity. Future prospective, randomized studies stratifying patients by renal risk factors are essential to more accurately assess the true renal effects of TAF and TDF [15, 16].

Our longitudinal analysis revealed temporal variations in lipid profile effects between TAF and TAF regimens. Significant lipid elevations with TAF were predominantly observed in the short-term analysis (Model 2: baseline to 24 months), with total cholesterol showing a between-group difference of -6.07 mg/dL (*p* = 0.001), non-HDL cholesterol − 5.22 mg/dL (*p* = 0.001), and triglycerides − 11.00 mg/dL (*p* = 0.010). Notably, these differences attenuated in the extended 48-month analysis (Model 1), suggesting potential metabolic adaptation or confounding factors over time. The temporal pattern of lipid changes may reflect TAF’s pharmacokinetic properties, including lower systemic tenofovir exposure with enhanced intracellular concentrations, which may influence hepatic lipid metabolism through mechanisms not yet fully elucidated [17, 18].

These findings have particular relevance for HIV treatment practices in Türkiye, where HIV cases have increased three-fold over the past decade [19]. Our results are included the 10 centers design across different geographical regions provides valuable data for developing national treatment protocols in line with Türkiye’s HIV/AIDS Control Program objectives [20].

According to our results, TAF was linked to greater short-term lipid increases and eGFR decline, while bone outcomes were broadly comparable to TDF; thus, we cannot claim superior renal or bone safety for TAF. Conversely, TDF’s more favorable lipid profile may benefit patients with metabolic syndrome or cardiovascular disease, despite its known renal and bone toxicity concerns [21, 22]. The utilization of mixed-effects regression modeling enhanced the robustness of our analysis by accounting for within-subject correlation, handling missing data efficiently, and providing unbiased estimates of treatment effects over time [23]. These findings underscore the necessity for individualized treatment approaches that balance efficacy, safety, and patient-specific risk factors in optimizing long-term outcomes for people living with HIV.

In clinical practice, the choice between TDF and TAF should be individualized based on patient age, comorbidities, and cardiovascular risk factors. TAF should be preferred in patients over 40 years of age, those with pre-existing kidney disease (eGFR < 60 mL/min/1.73 m²), hypertension, diabetes, high risk of osteoporosis, and postmenopausal women [24]. TAF is particularly recommended for individuals at risk of chronic kidney disease due to its superior safety profile. TDF may be preferred in younger patients (< 40 years), those with dyslipidemia or cardiovascular risk factors, when weight gain avoidance is desired, and when cost-effective treatment is required [24]. In patients with hepatitis B coinfection, both agents are effective; however, TDF is recommended for young patients with normal kidney function, while TAF is preferred for those with renal risk [25]. For elderly HIV patients (>50 years), TAF is more appropriate due to its bone and renal safety profile, although lipid profile and cardiovascular risk require close monitoring [26]. This individualized approach is critically important for optimizing long-term clinical outcomes. Our 48-month MER analysis revealed that despite comparable virological efficacy, the distinct safety profiles mandate personalized selection: TDF’s immunological and lipid advantages favor younger patients, while TAF’s superior renal and bone safety profile supports its use in patients with relevant comorbidities.

Exploratory subgroup analyses stratified by age (20–49 vs. 50 + years) and sex suggested potential differential treatment effects, though these findings require cautious interpretation given the post hoc nature and lack of multiple comparison corrections (Tables 3 and 4). Age-stratified analyses indicated possible trends toward differential renal outcomes, with younger patients (20–49 years) in the TAF group showing patterns that may suggest different eGFR trajectories compared to TDF over 48 months (*p* < 0.001). Similarly, bone mineral density changes appeared to vary by age group, with some indication of differential spine BMD preservation patterns in older patients (50 + years) at 24 months (*p* < 0.001). Sex-stratified analyses revealed heterogeneous patterns, with male participants showing potential differences in renal function trajectories (*p* < 0.001 at 48 months), while female participants demonstrated possible variations in bone health outcomes (spine BMD *p* = 0.042 at 48 months). Both treatment groups experienced lipid profile changes, with TAF-treated participants showing elevations in total cholesterol and non-HDL cholesterol levels (*p* ≤ 0.032). Additionally, gamma-glutamyl transferase (GGT) increases were observed in both sexes receiving TAF (males: *p* = 0.027; females: *p* = 0.033 at 24 months). While GGT elevations may indicate hepatic metabolic changes [27], the clinical significance of these biochemical alterations remains unclear without corresponding imaging or clinical endpoints, and these findings should not be interpreted as definitive evidence of hepatic steatosis. Antiviral efficacy remained comparable across all subgroups, with no clinically meaningful differences in CD4 + recovery or viral suppression rates. Given the exploratory nature of these subgroup analyses and the potential for chance findings due to multiple comparisons, these results should be considered hypothesis-generating rather than definitive. Future prospective studies with adequate power and pre-specified subgroup analyses are needed to confirm these preliminary observations and guide evidence-based treatment selection strategies.

While statistically significant differences were observed between TAF and TDF groups, the clinical significance requires interpretation through minimal clinically important differences (MCIDs) [28]. Established MCIDs suggest clinically meaningful changes of 15–20 mg/dL for total cholesterol/LDL and ≥ 25% eGFR decline [13, 29]. The clinical relevance of our observed differences may vary depending on individual patient risk profiles, emphasizing the importance of personalized treatment selection. Patients with cardiovascular risk factors may benefit from TDF despite TAF’s renal advantages, whereas those with renal or bone disease risk may warrant TAF selection. We believe that future studies should establish HIV-specific MCIDs and examine whether the observed differences translate into hard clinical endpoints.

### Limitations

This retrospective cohort study has several methodological constraints that affect interpretation. First, no a priori sample size calculation was performed; instead, all eligible patients (*n* = 540) were included. A post hoc power estimate suggested 84.36% power to detect small effects (f²=0.02, α = 0.05), but such analyses are prone to circularity and cannot substitute for prospective sample-size planning. Second, we lacked systematic documentation of concomitant medications with major metabolic effects (e.g., lipid-lowering agents such as statins or fibrates, antidiabetic therapies, and antihypertensives). Because these agents can modify lipid levels by amounts comparable to or greater than the between-group differences observed here, attribution of lipid changes to the antiretroviral regimens is not possible; accordingly, lipid findings should be considered preliminary and require confirmation in prospective studies with rigorous medication capture and control. Third, baseline imbalances and unmeasured confounding may have influenced results. We did not adjust for baseline values of each outcome, ART regimen components (e.g., bictegravir vs. elvitegravir/cobicistat), center effects, adherence, smoking, HBV/HCV coinfection, or concomitant medications affecting lipid or renal indices; thus, the analyses are susceptible to channeling bias and residual confounding. Although national HIV treatment guidelines were consistent across centers, we did not incorporate center-level adjustments. Fourth, age- and sex-stratified subgroup analyses were not prespecified and were conducted post hoc for exploratory purposes. No correction for multiple testing was applied; therefore, p values are exploratory, and some effect directions varied across time windows (e.g., 24 vs. 48 months), leading to potential inconsistencies. These subgroup findings should be interpreted with caution and should not be generalized without independent confirmation in adequately powered studies. Fifth, we did not correct for multiple comparisons overall; therefore, p values should be viewed as exploratory. Finally, temporal trends were modeled as linear across 0–48 months, an assumption that may not hold for all outcomes. In light of these limitations, results should be interpreted cautiously, and clinical decisions should not rely solely on the metabolic comparisons presented. Prospective studies with comprehensive measurement and control of confounders are needed to validate and extend these observations.

## Conclusion

In conclusion, while TDF and TAF-based regimens demonstrate comparable virological efficacy in HIV management, they exhibit significant differences in metabolic, biochemical, and bone health effects. TDF showed favorable lipid profile changes, whereas overall BMD was similar between groups; renal findings likely reflect channeling bias. These distinct safety profiles underscore the importance of individualized treatment selection based on patient-specific cardiovascular risk, renal function, and bone health status to optimize long-term clinical outcomes.

## Supplementary Information

Below is the link to the electronic supplementary material.

Supplementary Material 1

## Data Availability

The data supporting this study’s findings are available upon reasonable request from the corresponding author, Oguz Karabay. Additional data related to this study can be provided by contacting the corresponding author.
