# Supplementary material for: Long-term metabolic and safety profiles of tenofovir alafenamide and tenofovir disoproxil fumarate in ART-naive people living with HIV: a multicenter retrospective study using a mixed-effects model
Source: BMC Pharmacol Toxicol. 2025 Oct 14;26:164. doi: 10.1186/s40360-025-01003-0 (PMC12523089; doi:10.1186/s40360-025-01003-0)
Supplement: Supplementary file 1 — Supplementary Material 1 [file 40360_2025_1003_MOESM1_ESM.docx]

**Supplementary Table 1: Participating Centers and Patient Distribution**

| **Center** | **City** | **Patients (n)** | **Contributing Authors** |
| --- | --- | --- | --- |
| Sakarya University | Sakarya | 120 | OK, AV, İY, NCB, EG |
| Prof. Dr. Cemil Taşcıoğlu City Hospital | Istanbul | 40 | AK |
| Medipol University | Istanbul | 50 | RD |
| Istanbul Medeniyet University | Istanbul | 45 | YÇ, HÇ, MTŞ |
| Antalya Training and Research Hospital | Antalya | 50 | ZAO, UB |
| Haydarpaşa Numune Training and Research Hospital | Istanbul | 50 | SŞ |
| Kocaeli University | Kocaeli | 40 | SA, MD |
| Düzce University | Düzce | 45 | DY, Nİ, BT |
| Şanlıurfa Training and Research Hospital | Şanlıurfa | 50 | TDÇ |
| Ankara Bilkent City Hospital | Ankara | 50 | AB |
